# Diaphragm function and weaning from mechanical ventilation: an ultrasound and phrenic nerve stimulation clinical study

**DOI:** 10.1186/s13613-018-0401-y

**Published:** 2018-04-23

**Authors:** Martin Dres, Ewan C. Goligher, Bruno-Pierre Dubé, Elise Morawiec, Laurence Dangers, Danielle Reuter, Julien Mayaux, Thomas Similowski, Alexandre Demoule

**Affiliations:** 1 UPMC Univ Paris 06, INSERM, UMRS1158, Neurophysiologie Respiratoire Expérimentale et Clinique, Sorbonne Universités, Paris, France; 2 Service de Pneumologie et Réanimation Médicale (Département “R3S”), AP-HP, Groupe Hospitalier Pitié-Salpêtrière Charles Foix, 47-83 boulevard de l’Hôpital, 75013 Paris, France; 3 Interdepartmental Division of Critical Care Medicine, University of Toronto, Toronto, Canada; 4 Division of Respirology, Department of Medicine, University Health Network and Mount Sinai Hospital, Toronto, Canada; 5 Département de Médecine, Service de Pneumologie, Hôpital Hôtel-Dieu, Centre Hospitalier de l’Université de Montréal (CHUM), Montréal, QC Canada

**Keywords:** Liberation, Ventilator, Diaphragm, Weakness, Ultrasound, Extubation

## Abstract

**Background:**

Diaphragm dysfunction is defined by a value of twitch tracheal pressure in response to magnetic phrenic stimulation (twitch pressure) amounting to less than 11 cmH_2_O. This study assessed whether this threshold or a lower one would predict accurately weaning failure from mechanical ventilation. Twitch pressure was compared to ultrasound measurement of diaphragm function.

**Methods:**

In patients undergoing a first spontaneous breathing trial, diaphragm function was evaluated by twitch pressure and by diaphragm ultrasound (thickening fraction). Receiver operating characteristics curves were computed to determine the best thresholds predicting failure of spontaneous breathing trial.

**Results:**

Seventy-six patients were evaluated, 48 (63%) succeeded and 28 (37%) failed the spontaneous breathing trial. The optimal thresholds of twitch pressure and thickening fraction to predict failure of the spontaneous breathing trial were, respectively, 7.2 cmH_2_O and 25.8%, respectively. The receiver operating characteristics curves were 0.80 (95% CI 0.70–0.89) for twitch pressure and 0.82 (95% CI 0.73–0.93) for thickening fraction. Both receiver operating characteristics curves were similar (*p* = 0.83). A twitch pressure value lower than 11 cmH_2_O (the traditional cutoff for diaphragm dysfunction) predicted failure of the spontaneous breathing trial with a sensitivity of 89% (95% CI 72–98%) and a specificity of 45% (95% CI 30–60%).

**Conclusions:**

Failure of spontaneous breathing trial can be predicted with a lower value of twitch pressure than the value defining diaphragm dysfunction. Twitch pressure and thickening fraction had similar strong performance in the prediction of failure of the spontaneous breathing trial.

**Electronic supplementary material:**

The online version of this article (10.1186/s13613-018-0401-y) contains supplementary material, which is available to authorized users.

## Background

Diaphragm dysfunction is common in critically ill patients exposed to mechanical ventilation [1]. It can occur soon after intubation [2]. It can also occur later, where it may be a consequence of intensive care unit acquired weakness or the result of the specific time-dependent impact of mechanical ventilation on the diaphragm [3–7], a phenomenon referred to as ventilator-induced diaphragm dysfunction [8]. Diaphragm dysfunction is associated with increased mortality [2, 3, 9] and delayed liberation from mechanical ventilation [3, 4, 10, 11].

Diaphragm dysfunction manifests as a reduced capacity to generate inspiratory pressure and flow [12]. This can be assessed in term of the negative pressure swing measured at the opening of an endotracheal tube in response to bilateral phrenic nerve stimulation (Ptr,stim) [1]. Outside of the intensive care context, a Ptr,stim value amounting to less than 11 cmH_2_O is considered indicative of diaphragm dysfunction [12–14]. In critically ill patients, this value of − 11 cmH_2_O has proven useful from a prognostic point of view. In a prospective study of ICU patients in whom Ptr,stim was measured at time of weaning, patients with a Ptr,stim below the 11 cmH_2_O threshold were less likely to survive to discharge from the ICU or hospital than those with a Ptr,stim above this threshold [3]. Yet, lower Ptr,stim values are commonly encountered in ICU patients at various points of their ICU stay [2–4, 15] and two recent studies have reported successful weaning from mechanical ventilation despite lower values of Ptr,stim [3, 4]. Therefore, our hypothesis was that the Ptr,stim threshold value used to define diaphragm dysfunction (− 11 cmH_2_O) would be not necessarily the best threshold that allows successful or failed weaning from mechanical ventilation. The present study was designed to identify the optimal Ptr,stim value to predict failure of the spontaneous breathing trial. In view of the recently reported utility of diaphragm thickening fraction (TFdi) [16] to predict failure of the spontaneous breathing trial, the predictive value of this variable was also evaluated.

## Patients and methods

This study was an ancillary analysis of a study prospectively conducted over 9 months (November 1, 2014, to July 31, 2015) in a medical 10-bed ICU. Human research ethics committee approval for the study was provided by the Comité de Protection des Personnes—Ile de France 6 (ID RCB: 2014-A00715-42). Informed consent was obtained from all patients or their relatives. Data from this cohort have been previously published [3, 17].

### Patients

Patients were eligible for inclusion if they had been intubated and ventilated for at least 24 h and if they met predefined readiness-to-wean criteria on daily screening [18] and were therefore ready for a first spontaneous breathing trial (Additional file 1: readiness criteria to initiate a spontaneous breathing trial). Readiness-to-wean criteria were searched for while patients were ventilated on existing mechanical ventilation setting prior to spontaneous breathing trial (SBT). Patients with clinical factors potentially interfering with phrenic nerve stimulation, who had a tracheostomy, or who were unable to follow simple orders were excluded (Additional file 1: exclusion criteria).

### Measurements

All measurements were taken a few minutes before starting the SBT. Phrenic nerve stimulation was performed while patients were briefly disconnected from the ventilator (Additional file 1: description of the phrenic nerves stimulation technique), and diaphragm ultrasound (Additional file 1: description of the ultrasound technique) was conducted while patients were mechanically ventilated under pressure support ventilation with ventilator settings decided by the attending physician. In our unit, pressure support level is set in order to provide a tidal volume of 6–8 ml/kg of ideal body weight without any sign of acute respiratory distress or discomfort. Positive end-expiratory pressure is set at 5 cmH_2_O.

Diaphragm function was assessed in terms of changes in tracheal pressure during a magnetic stimulation (Ptr,stim), as described elsewhere [2, 4, 5, 14, 15]. Stimulations were delivered at the maximum intensity allowed by the stimulator (100%) known to result in supramaximal diaphragm contraction in most patients [2, 10, 13, 15, 19]. Diaphragm ultrasound was conducted using a 4–12-MHz linear array transducer (Sparq ultrasound system, Philips, Philips Healthcare, MA, USA). Diaphragm thickness was measured at end-expiration (Tdi,ee) and end-inspiration (Tdi,ei), and thickening fraction (TFdi) was calculated offline as (Tdi,ei–Tdi,ee)/Tdi,ee. Two observers blinded to the results of phrenic nerve stimulation performed diaphragm ultrasound. As previously reported elsewhere [3], the reproducibility of ultrasound measurements was assessed on the first 20 patients while the two observers were blinded to each other’s measurements and after they performed at least 20 diaphragm ultrasounds during a 2-month training period before starting the study [3, 17]. Intra-class correlation (ICC) for Tdi,ei, Tdi,ee and TFdi were, respectively: ICC = 0.95 (*p* < 0.001), ICC = 0.96 (*p* < 0.001) and ICC = 0.87 (*p* < 0.001) [3].

### Study design

After obtaining study measurements, patients underwent a SBT. During the SBT, patients were ventilated with a pressure support level 7 cmH_2_O and 0 cmH_2_O end-expiratory pressure for 30 min. Failure of the SBT was defined if patients developed criteria for clinical intolerance defined as follows [18]: (1) pulsed oxygen saturation < 90% with a fraction of inspired oxygen ≥ 50%, acute respiratory distress (respiratory rate ≥ 40/min with agitation or cyanosis), systolic arterial blood pressure ≥ 180 mmHg, or pH < 7.32 with an arterial carbon dioxide tension ≥ 50 mmHg. For patients with multiple failed SBT, only their first SBT was considered for the analysis.

### Statistical analysis

Continuous variables are expressed as median (interquartile range), and categorical variables are expressed as absolute and relative frequency. Continuous variables were compared with Mann–Whitney *U* test.

The manuscript conforms to the STARD checklist for reporting of studies of diagnostic accuracy [20]. Receiver operating characteristic (ROC) curves were constructed to evaluate the performance of the two index to predict SBT failure: Ptr,stim and TFdi. Sensitivities, specificities, positive and negative predictive values, positive and negative likelihood ratios and areas under the ROC curves (AUC-ROC) were calculated. AUC-ROC were performed to identify optimal cutoff values of Ptr,stim and TFdi in predicting SBT failure, and these estimates were obtained using bootstrapping with 1000 replications. The best threshold value for each index was determined as the value associated with the best Youden index for the prediction of SBT failure. AUC-ROC were compared using the nonparametric approach of DeLong et al. [21].

For all final comparisons, a two-tailed *p* value less than or equal to 0.05 was considered statistically significant. Statistical analyses were performed with MedCalc (MedCalc Software bvba).

## Results

Between November 1, 2014, and July 31, 2015, 330 patients were admitted in our ICU. One hundred and eighty-four patients received invasive mechanical ventilation for more than 24 h leading to the enrollment of 76 consecutive patients in the study (Additional file 1: Figure E1. Flowchart of the study). The characteristics of these patients upon inclusion are given in Table 1.

**Table 1:** Patient’s characteristics at inclusion

Characteristics	
Female, *n* (%)	24 (32)
Age, years	58 (48–68)
SOFA	5 (4–7)
Duration of mechanical ventilation, days	4 (2–6)
Main reason for mechanical ventilation, *n* (%)	
Acute respiratory failure	28 (37)
Shock	24 (32)
Coma	23 (31)
Ventilator parameters	
Pressure support level, cmH_2_O	10 (8–10)
Tidal volume, ml/kg ideal body weight	7 (5–8)
PEEP, cmH_2_O	5 (5–6)
Clinical parameters	
Breaths, min^−1^	22 (20–25)
Mean arterial pressure, mmHg	80 (69–98)
Heart rate, min^−1^	89 (78–100)
Arterial blood gases	
pH	7.44 (7.40–7.45)
PaCO_2_, mmHg	38 (34–44)
PaO_2_/FiO_2_	279 (214–357)

Continuous variables are expressed as median (interquartile range), and categorical variables are expressed as absolute value (%)

*SOFA* sequential organ failure assessment, *PEEP* positive end-expiratory pressure, *PaO*_*2*_*/FiO*_*2*_ ratio of arterial oxygen tension to inspired oxygen fraction

Forty-eight patients (63%) passed the SBT and were subsequently extubated, while 28 patients (37%) developed criteria for SBT failure and initial ventilator settings were accordingly resumed. Of the 48 extubated patients, seven patients required resumption of ventilatory support (six were reintubated and 1 had curative noninvasive ventilation) within 48 h: five patients for respiratory distress and two patients for loss of consciousness. No stridor was reported. Prophylactic noninvasive ventilation was used in two patients.

### Prediction of spontaneous breathing trial failure

**Fig. 1:**
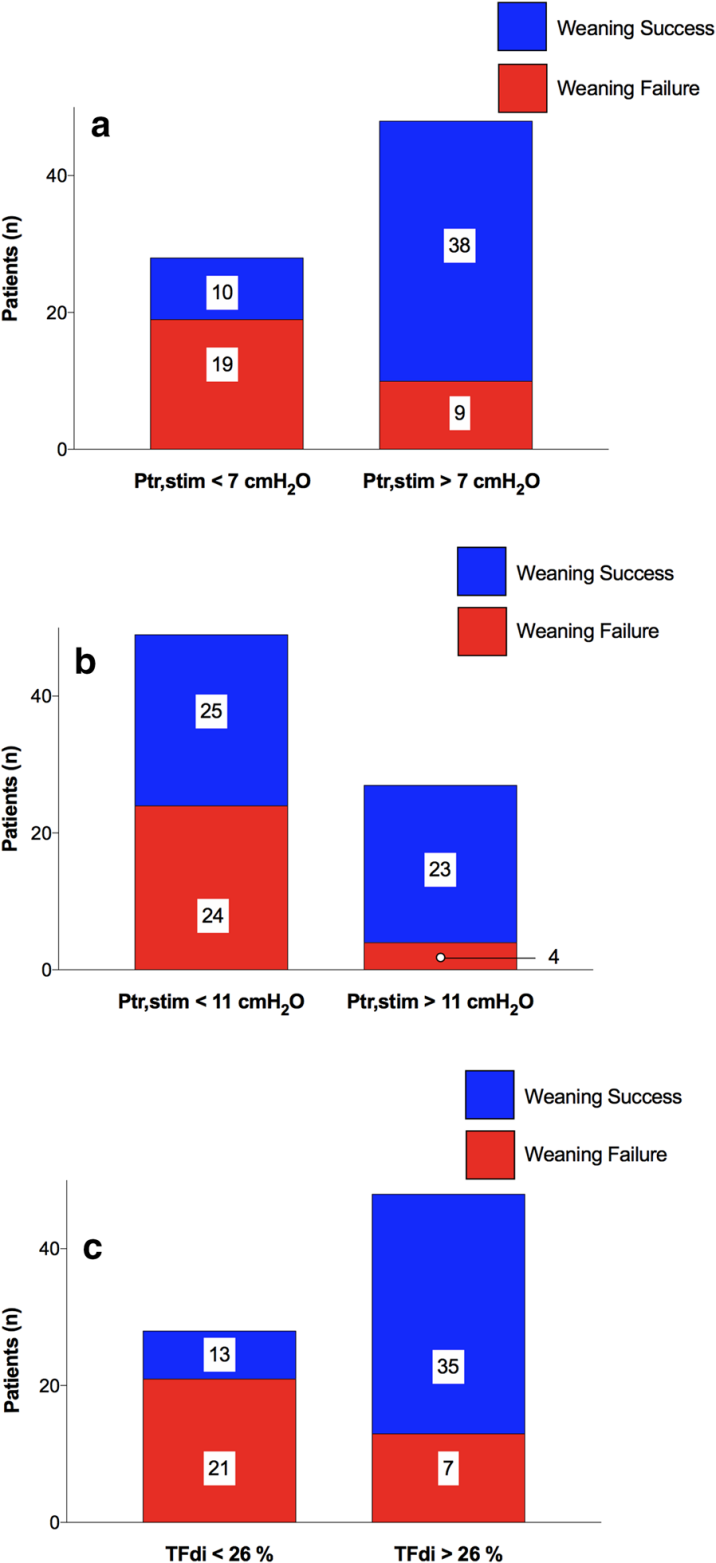
Patients with successful spontaneous breathing trial and failed spontaneous breathing trial according to 7 cmH_2_O (**a**) and 11 cmH_2_O (**b**) thresholds of endotracheal pressure induced by a bilateral phrenic nerve stimulation (Ptr,stim) and 26% (**c**) threshold of diaphragm thickening fraction (TFdi). Numbers indicate the number of patients in each category

Median Ptr,stim was 8.2 (5.9–12.6) cmH_2_O; Ptr,stim was 10.0 (7.3–14.3) and 6.5 (3.0–8.8) cmH_2_O in patients with successful and failed SBT, respectively (*p* < 0.001). The optimal threshold value of Ptr,stim to predict SBT failure was 7.2 cmH_2_O (Table 2). A Ptr,stim value lower than 11 cmH_2_O (the traditional cutoff for diaphragm dysfunction) predicted SBT failure with a sensitivity of 89% (95% CI 72–98%) and a specificity of 45% (95% CI 30–60%). Patients with SBT success and SBT failure according to both 7.0 and 11.0 cmH_2_O thresholds of Ptr,stim are shown in Fig. 1a, b.

**Table 2:** Threshold, area under the receiver operating characteristics curves (AUC-ROC), sensitivity, specificity, positive and negative likelihood ratios and positive and negative predictive values of endotracheal pressure induced by a bilateral phrenic nerve stimulation (Ptr,stim) and diaphragm thickening fraction (TFdi) to predict weaning failure from mechanical ventilation

	Threshold	AUC-ROC (95% CI)	Sensitivity (%) (95% CI)	Specificity (%) (95% CI)	Likelihood ratios (95% CI)		Predictive values (%) (95% CI)	
					Positive	Negative	Positive	Negative
Ptr,stim	7.2 cmH_2_O	0.80 (0.70–0.89)	68 (47–84)	79 (64–89)	3.2 (1.7–5.8)	0.4 (0.2–0.7)	66 (51–78)	80 (70–88)
TFdi	25.8%	0.82 (0.73–0.93)	79 (59–92)	73 (58–85)	2.9 (1.8–4.8)	0.3 (0.1–0.6)	63 (51–74)	85 (74–92)

*CI* confidence interval

**Fig. 2:**
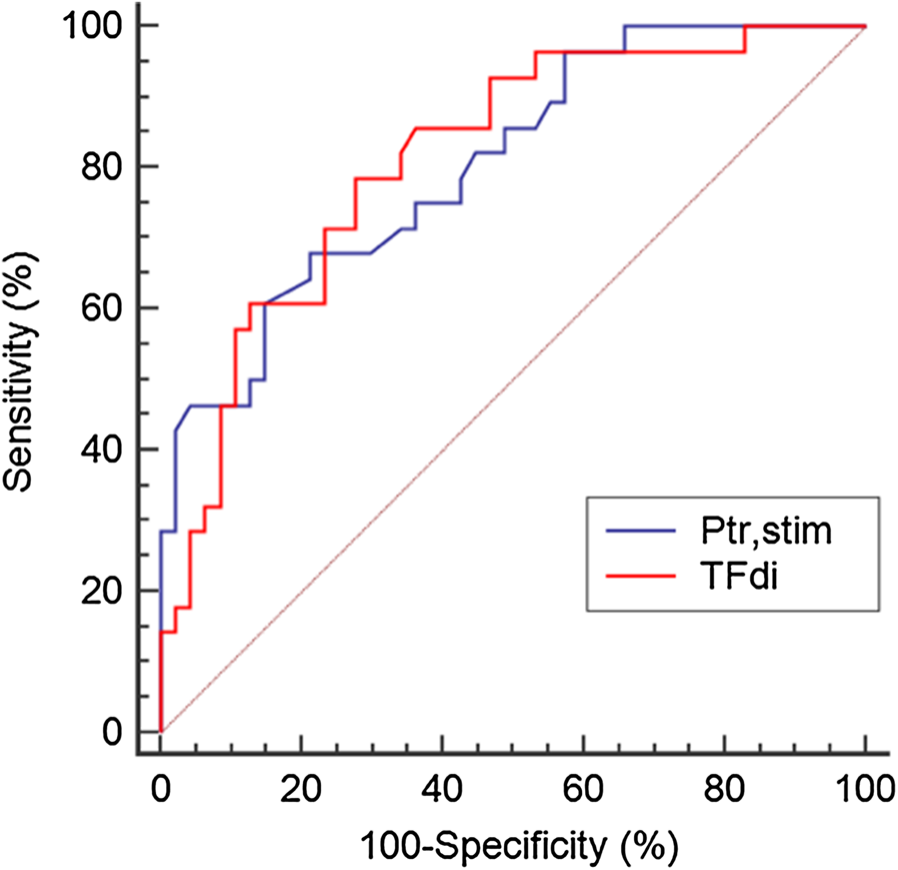
Receiver operating characteristics curves of endotracheal pressure induced by a bilateral phrenic nerve stimulation (Ptr,stim) and diaphragm thickening fraction (TFdi) to predict failure of the spontaneous breathing trial

Median TFdi was 28% (19–35) in the whole population; TFdi was 33% (29–43) and 19% (11–25) in patients with successful SBT and SBT failure, respectively (*p* < 0.001). The optimal threshold value of TFdi to predict SBT failure was 25.8%. Figure 1c shows the number of patients with SBT success and SBT failure according to 25.8%-TFdi threshold. Predictive performances of TFdi are shown in Table 2. The comparison of AUC-ROC of Ptr,stim and TFdi is displayed in Fig. 2. Ptr,stim and TFdi had similar AUC-ROC (*p* = 0.83).

## Discussion

This study reports a dual assessment of diaphragm function and its relationship with weaning outcome in mechanically ventilated medical patients undergoing a first spontaneous breathing trial. Our findings can be summarized as follows: (1) a lower value of Ptr,stim (i.e., 7.0 cmH_2_O) than the value commonly accepted value to define diaphragm dysfunction (i.e., 11.0 cmH_2_O) is more reliable to predict SBT failure, (2) Ptr,stim and TFdi are equivalent to predict SBT failure.

### Diaphragm function and weaning from mechanical ventilation

The negative impact of diaphragm dysfunction on successful weaning from mechanical ventilation has been established by several investigations in critically ill patients [3, 4, 11, 22]. At the time of weaning, diaphragm dysfunction is highly prevalent [1] with reported rates ranging from 25–30% [11, 22] to 60–80% [3, 4]. To our knowledge, only three studies have assessed diaphragm dysfunction at the time of attempted liberation from mechanical ventilation using the gold standard technique, namely the phrenic nerves stimulation [3, 4, 23]. However, none of them provided any threshold values for Ptr,stim to predict weaning outcome. Of note, these studies including ours indicate that a substantial proportion of patients (up to 44%) can be successfully weaned from the ventilator despite having diaphragm dysfunction defined as Ptr,stim < 11 cmH_2_O [3, 4]. Therefore, *normal* diaphragm function according to a definition established in healthy subjects [12] is not a prerequisite for a successful SBT. This finding is not altogether surprising as many patients with chronic diaphragm dysfunction do not require mechanical ventilation [24, 25]. While diaphragm dysfunction might limit exercise capacity, the clinical consequences of diaphragm dysfunction in successfully liberated patients are uncertain. However, the impact of respiratory muscles dysfunction (not specifically the diaphragm) after critical illness may be of importance since it is associated with worse long-term outcomes [26, 27]. Overall, our findings are of importance since they highlight that presence of diaphragm dysfunction at the time of weaning should not discourage clinicians from attempting liberation from ventilation. By contrast, not all patients (23/27) with a Ptr,stim higher than 11.0 cmH_2_O had a successfully SBT. As a matter of fact, the 11.0 cmH_2_O threshold of Ptr,stim was associated with a lower specificity but a higher sensitivity than the 7.0 cmH_2_O threshold in the prediction of SBT failure. The lower 7.0 cmH_2_O Ptr,stim threshold provides the optimal combination of sensitivity and specificity in the prediction of SBT failure.

### Diaphragm ultrasound in the prediction of SBT failure

The use of diaphragm ultrasound is growing in the ICU [28, 29]. It has many advantages over phrenic nerve stimulation, which requires costly equipment and extensive technical expertise. Ultrasound is a noninvasive and highly feasible bedside imaging modality, and ultrasound devices are widely available in ICUs. Several studies have proposed various ultrasound-derived markers aiming at assessing diaphragm function. Importantly, in our study, Ptr,stim and TFdi demonstrated similar performance in the prediction of weaning. Of note, the optimal TFdi cutoff (26%) identified in our study is very close to the cutoffs reported in previous investigations [16, 30, 31]. Considering ultrasound as a substitute of the phrenic nerves stimulation technique, it will make diaphragm evaluation much easier at the bedside. However, the indication of diaphragm ultrasound during the weaning process is not yet clearly defined. In addition, it is important to remind that the majority of patients are shortly and safely separated from the ventilation. As it happens, the place of diaphragm ultrasound might be viewed as a complementary investigation and not as a surrogate of clinical judgment. It may be used as a screening tool to identify patients who are at high risk of SBT failure (before conducting the SBT) or as a diagnostic method to determine the cause of SBT or extubation failure [32].

### Strengths and limitations of our study

This study is the largest to report a dual approach providing comparison between the gold standard evaluation method of diaphragm function and diaphragm ultrasound during the weaning phase. However, this study has limitations. First, the generalizability of our findings may be limited by the characteristics of the patients of our cohort. Accordingly, our study might be viewed as a hypothesis generator and further trials are warranted to confirm the clinical relevance of our findings. Second, while we obtained good inter- and intra-reproducibility in the measurements of diaphragm ultrasound, centers employing the technique must also demonstrate adequate technical skill (based on reproducibility) before implementing the technique for clinical purposes. Third, we performed diaphragm ultrasound while patients were ventilated with pressure support and not during the SBT. While this approach is easier to implement (no change in ventilator setting) and less stressful for patients, it could underestimate diaphragm thickening [33]. However, the amount of pressure support was standardized in order to target a tidal volume between 6 and 8 ml/kg predicted body weight. Reassuringly, any effect of ventilatory support on TFdi is likely to introduce ‘noise’ in its correlation with weaning outcome and this would tend to bias the observed association toward the null. Fourth, we have assessed diaphragm function by using the changes in tracheal twitch pressure rather than the changes in transdiaphragmatic twitch pressure. This last measurement is more specific to the diaphragm function but requires the placement of two balloons, which make it more invasive. Although the two twitch pressures are not interchangeable, they are well correlated [15].

## Conclusions

Diaphragm ultrasound is a reliable surrogate of the phrenic nerve stimulation method in the assessment of diaphragm function to predict weaning outcome. A multicenter investigation is now required to confirm whether the 26% value of TFdi cutoff could or could not be used widely to predict SBT outcome. Diaphragm ultrasound could be combined with cardiac echo or lung ultrasound to tailor post-extubation management according to the risk of weaning failure. Although diaphragm dysfunction did not systematically impair weaning outcome, it may behave as a marker of severity and poor prognosis. Future studies should address this hypothesis and investigate mid- and long-term consequences of diaphragm dysfunction on patient functional status and quality of life.

## Additional file

**Additional file 1.** Full description of the Methods. **Figure E1.** Flow chart of the patients.

## Abbreviations

SBT: Spontaneous breathing trial
Ptr,stim: Tracheal pressure in response to bilateral magnetic stimulation of the phrenic nerves
TFdi: Diaphragm thickening fraction
ICU: Intensive care unit
AUC-ROC: Area under the curve of receiving operating characteristics
