# Supplementary material for: Diaphragm function and weaning from mechanical ventilation: an ultrasound and phrenic nerve stimulation clinical study
Source: Ann Intensive Care. 2018 Apr 23;8:53. doi: 10.1186/s13613-018-0401-y (PMC5913054; doi:10.1186/s13613-018-0401-y)
Supplement: Supplementary file 1 — Additional file 1. Full description of the Methods. Figure E1. Flow chart of the patients. [file 13613_2018_401_MOESM1_ESM.docx]

**Diaphragm function and liberation from mechanical ventilation:**

**An ultrasound and phrenic nerve stimulation clinical study**

Martin Dres, Ewan Goligher, Bruno-Pierre Dubé, Elise Morawiec, Laurence Dangers, Danielle Reuter, Julien Mayaux, Thomas Similowski, Alexandre Demoule

***Additional file 1***

**Methods**

***Readiness criteria to initiate a spontaneous breathing trial (SBT)***

A SBT was initiated as soon as all the four following criteria were present [1]:

1. adequate oxygenation as stated by a SpO2 > 90 % on a fraction of inspired oxygen (FiO2) ≤ 40 % and positive end expiratory pressure (PEEP) ≤ 8 cmH_2_O,
2. adequate pulmonary function as stated by a respiratory rate ≤ 35/min,
3. a cooperative cognitive state,
4. stable cardiovascular status as stated by a systolic arterial blood pressure of 90-160 mmHg without or minimal vasopressors and heart rate ≤140/min.

***Exclusion criteria***

Exclusion criteria were related to any contra-indications or impossibility to perform magnetic stimulation of the phrenic nerves among the following: cardiac pacemaker or implanted defibrillator, cervical implants, use of neuromuscular blocking agents within the 24 hours preceding the diaphragm function assessment (with the exception of succinylcholine used during rapid-sequence induction of anaesthesia for intubation), pre-existing neuromuscular disorders, factors possibly interfering with tracheal pressure measurements in response to phrenic stimulation (multiple functioning chest drains, intrinsic positive end expiratory pressure). Intrinsic positive end expiratory pressure was found when at relaxed end-expiration, the endotracheal pressure could not reach the zero baseline while the endotracheal tube was disconnected from the ventilator, manually occluded and by checking the absence of respiratory effort. Finally, age less than 18 years, known pregnancy, and a decision to withhold life-sustaining treatment were also exclusion criteria.

***Criteria defining SBT failure***

The presence of at least one of the five following criteria defined SBT failure [1]:

1. blood oxygen saturation (SpO_2_) of < 90 % with a fraction of inspired oxygen (FiO_2_) ≥ 50 %,
2. acute respiratory distress (RR ≥ 40/min, agitation, cyanosis),
3. systolic arterial blood pressure ≥ 180 mmHg or increase by ≥ 20 %,
4. heart rate ≥ 140/min or increase by ≥ 20 %,
5. respiratory acidosis defined as pH < 7.32 with an arterial carbon dioxide tension (PaCO_2_) ≥ 50 mmHg.

If none of these failure criteria was present, the SBT was considered as successfully completed and the patient was extubated. The decision was ultimately taken by the attending physician.

***Description of the phrenic nerves stimulation technique***

Diaphragm pressure generating capacity was assessed in terms of the changes in endotracheal tube pressure induced by bilateral phrenic nerve stimulation during airway occlusion (Ptr,stim) as it has been already described elsewhere [2–4]. Phrenic nerve stimulation was performed by bilateral anterior magnetic stimulation. Briefly, two figure-of-eight coils connected to a pair of Magstim® 200 stimulators (The Magstim Company, Dyfed, UK) were positioned immediately posterior to the sternomastoid muscles at the level of the cricoid cartilage. Stimulations were delivered at the maximum output intensity of the stimulator (100%) that is known to result in supramaximal phrenic contraction [3,5–7]. The patients were studied in a standardized semi-recumbent position, as follows: end-expiratory pressure was set to zero and the patient was allowed to exhale during an end-expiratory pause. While the endotracheal tube was manually occluded, bilateral anterolateral magnetic stimulation was performed. The absence of active respiratory efforts in response to stimulation was determined by checking the stability of the airway pressure signal. Two operators were required to achieve both stimulation and measurements. After positioning the coils, at least three stimulations were performed at 100% of maximal output allowed by the stimulator. Stimulations were separated by at least 60-sec to avoid superposition. The average of three measures was taken into account for analysis. Ptr,stim was defined as the amplitude of the negative pressure wave following stimulation, taken from baseline to peak. It was measured at the proximal external end of the endotracheal tube, using a linear differential pressure transducer (MP45 ± 100 cmH_2_O, Validyne, Northridge, Calif., USA). The pressure signal was sampled and digitized at 100 Hz (MP30, Biopac Systems, Santa Barbara, Calif., USA or Powerlab, AD Instruments, Bella Vista, Australia) for subsequent data analysis.

***Description of the diaphragm ultrasound technique***

Ultrasound measurements were performed by two investigators after a 2 months training session in diaphragmatic ultrasound. Inter-observer reliability of the ultrasound measurements has been described elsewhere [8].

Ultrasound assessment of the diaphragm thickening was performed using a 4-12 MHz linear array transducer (Sparq ultrasound system, Phillips, Philips Healthcare, Andover, MA, USA). As previously reported [9,10], the probe was placed perpendicular to the right chest wall, at the midaxillary line between the 9^th^ and 10^th^ right intercostal spaces (at the level of the zone of apposition) and the right diaphragm was identified as a three-layered structure comprising two hyperechoic lines representing the pleural and peritoneal membranes and an middle hypoechoic layer representing the diaphragmatic muscle fibers. Using M-mode at a sweep speed of 10 mm/s, at least three spontaneous quiet breathing cycles were recorded and the image was frozen. Diaphragm thickness was measured at end-expiration (Tdi,ee) and end-inspiration (Tdi,ei) using electronic calipers. The thickening fraction of the diaphragm (TFdi) was calculated offline as (Tdi,ei – Tdi,ee)/ Tdi,ee.

For all measurement, at least three valid breathing cycles were recorded, and the average of the individual values was reported. Ultrasounds were performed by one of the investigators (either B.P.D or M.D.).

**Results**

**Figure E1.** Flow chart of the study.

**References**

1. Boles J-M, Bion J, Connors A, Herridge M, Marsh B, Melot C, et al. Weaning from mechanical ventilation. Eur. Respir. J. Off. J. Eur. Soc. Clin. Respir. Physiol. 2007;29:1033–56.

2. American Thoracic Society/European Respiratory Society. ATS/ERS Statement on respiratory muscle testing. Am. J. Respir. Crit. Care Med. 2002;166:518–624.

3. Demoule A, Jung B, Prodanovic H, Molinari N, Chanques G, Coirault C, et al. Diaphragm dysfunction on admission to the intensive care unit. Prevalence, risk factors, and prognostic impact-a prospective study. Am. J. Respir. Crit. Care Med. 2013;188:213–9.

4. Mills GH, Ponte J, Hamnegard CH, Kyroussis D, Polkey MI, Moxham J, et al. Tracheal tube pressure change during magnetic stimulation of the phrenic nerves as an indicator of diaphragm strength on the intensive care unit. Br. J. Anaesth. 2001;87:876–84.

5. Supinski GS, Callahan LA. Diaphragm weakness in mechanically ventilated critically ill patients. Crit. Care Lond. Engl. 2013;17:R120.

6. Mills GH, Kyroussis D, Hamnegard CH, Polkey MI, Green M, Moxham J. Bilateral magnetic stimulation of the phrenic nerves from an anterolateral approach. Am. J. Respir. Crit. Care Med. 1996;154:1099–105.

7. Watson AC, Hughes PD, Louise Harris M, Hart N, Ware RJ, Wendon J, et al. Measurement of twitch transdiaphragmatic, esophageal, and endotracheal tube pressure with bilateral anterolateral magnetic phrenic nerve stimulation in patients in the intensive care unit. Crit. Care Med. 2001;29:1325–31.

8. Dres M, Dubé B-P, Mayaux J, Delemazure J, Reuter D, Brochard L, et al. Coexistence and Impact of Limb Muscle and Diaphragm Weakness at Time of Liberation from Mechanical Ventilation in Medical Intensive Care Unit Patients. Am. J. Respir. Crit. Care Med. 2017;195:57–66.

9. Goligher EC, Laghi F, Detsky ME, Farias P, Murray A, Brace D, et al. Measuring diaphragm thickness with ultrasound in mechanically ventilated patients: feasibility, reproducibility and validity. Intensive Care Med. 2015;41:642–9.

10. Matamis D, Soilemezi E, Tsagourias M, Akoumianaki E, Dimassi S, Boroli F, et al. Sonographic evaluation of the diaphragm in critically ill patients. Technique and clinical applications. Intensive Care Med. 2013;39:801–10.
